# A Comprehensive Evaluation of China's TCM Medical Service System: An Empirical Research by Integrated Factor Analysis and TOPSIS

**DOI:** 10.3389/fpubh.2020.532420

**Published:** 2020-09-29

**Authors:** Zhi-Guang Li, Hua Wei

**Affiliations:** Department of Economics, School of Economics and Management, Anhui University of Chinese Medicine, Hefei, China

**Keywords:** traditional Chinese medicine, Healthy China 2030, health care system, medical reform, factor analysis, TOPSIS

## Abstract

**Objectives:** This paper constructs a comprehensive evaluation index of the traditional Chinese medicine (TCM) medical service system and summarizes the development of TCM medical services in China.

**Methods:** We chose 31 provinces' TCM hospitals as research objects. The data were obtained from the Health Statistics Yearbook from 2013 to 2018 and from the National Statistics of Chinese Medicine from 2012 to 2017. The approaches to factor analysis and TOPSIS are used in this paper. It is found that the comprehensive evaluation indexes of the TCM medical service system can be divided into 4 first-level indicators and 14 second-level indicators.

**Results:** The development of the TCM medical service system in China is unbalanced and inadequate. North China and East China are generally superior to Northwest and Southwest China in terms of revenue and expenditure for TCM medical services. The per capita of medical resources in the Southwest and Northwest are stronger than those in Central and South China, but overall medical resources are weaker than those in East China and North China. TCM medical service institutions in East China, South China and Central China have achieved better service results and higher economic benefits with less resource input, which further indicates the efficient allocation of resources and the balanced operation of TCM medical service institutions.

**Conclusion:** The development of China's TCM medical service system shows the imbalance and inadequacy of “East is strong, West is weak” and “South is superior, North is inferior.”

## Introduction

In June 2016, the Chinese government created a program of public policies within its planning called “Healthy China 2030.” A key issue that the outline of the Healthy China 2030 Plan involves is to highlight the unique advantages of traditional Chinese medicine (TCM), especially in disease prevention. There is evidence that TCM, with its unique advantages in health care, can be recognized as one of the most important treatments in the world. Therefore, the Chinese government has elevated the development of TCM as a national strategy (1). The Chinese health care system and its reform has long attracted international attention and achieved great success, although challenges remain (2, 3). In the last few decades, Western medicine has dominated in China, although traditional Chinese medicine still plays an important role in the Chinese health care system (4). In light of recent events in Chinese new medical reform, construction of the TCM medical service system is emphasized not only by the government, but also by individual TCM hospitals and various departments inside hospitals. Because TCM medical resource allocation is extremely unbalanced and inadequate, especially between regions and between urban and rural areas (5). That is to say, the balance and coordination of related resources should be a priority in shaping China's health-care system reform (6). Therefore, how to establish a Chinese TCM medical service system and meet the national demand for TCM services has become a major concern of stakeholders, such as TCM hospitals and local authorities (7). In fact, the construction of a high-quality, efficient TCM medical service system entails not only the upgrading and transformation of the existing health care system, but also comprehensively deepening reform and promoting the implementation of China's health strategy (8).

The TCM medical service system refers to the TCM health resources of TCM medical institutions and other medical institutions, which are formed in the process of providing TCM medical services (9). As the average resources of TCM medical institutions are relatively weak, development is uneven, and their service functions are limited, which further indicates that improving the TCM medical service system in China is the primary task for the development of TCM (10, 11). Only if the evaluation indexes of the TCM medical service system are determined, can we develop the TCM medical service correctly and reasonably. In fact, the comprehensive evaluation of TCM medical services also plays a role in promoting the development of TCM hospitals, the direction of hospital management, and the internal management of hospitals (12).

At the time of the current study, the construction of the TCM medical service system is still in the exploratory stage, and people have not reached a consensus on a comprehensive evaluation mechanism of the TCM service system. Gu constructed a comprehensive evaluation system of clinical departments with medical quality, work efficiency, sustainable development of departments, social benefits, and economic management as first-level indicators (13). Ni and Wang et al. took social responsibility as the starting point by using the information analysis method and Delphi expert consultation method to explore the establishment of three-level public hospitals' public welfare evaluation index system, which mainly includes three first-level indicators of service quality, service suitability, and professional ethics (14, 15). Liu also completed a comprehensive evaluation of the medical service capacity of state-owned hospitals by using the expert consultation method, including three first-level indicators, namely resource allocation, service capacity and medical technology (16). Only Zhou focused on traditional Chinese medicine hospitals and constructed a comprehensive evaluation model of TCM medical service quality, including seven first-level indicators, namely TCM technicians, TCM infrastructure, TCM revenue and expenditure, TCM medical technology, TCM services, operating efficiency and patient satisfaction (17). Although some research has been carried out on the TCM medical service system, there have been no empirical investigations into explaining the logical relationship between the indicators and the weight in the TCM medical service system. Additionally, previous studies have suffered from methodological flaws and limitations. For example, the Delphi method and expert consultation method are not exempt from significant methodological weaknesses, such as their basic source of information (who can be defined as an expert, what biases each expert has, etc.), the use of consensus as a way to approach the truth, the limitation of the interaction involved in written and controlled feedback, and the restriction to the possibility of social compensation for individual contributions to the group (18). However, the methodological approach taken in this study is a mixed methodology based on integrated Factor Analysis and TOPSIS. Finally, we are going to assess the trends and development of TCM medical services in 31 provinces of China.

**Figure 1:**
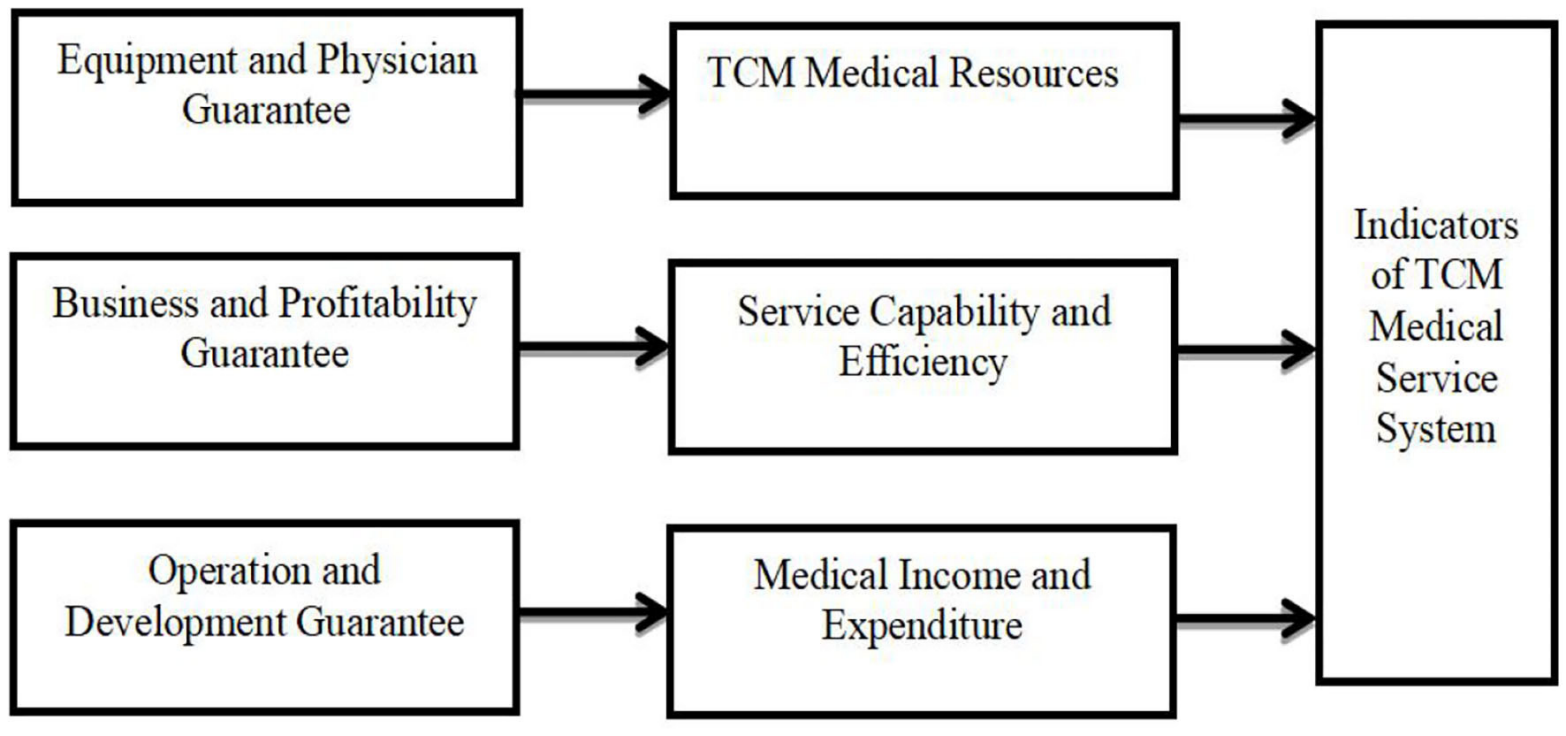
Framework of the TCM medical service system.

In this context, the resource-based view (RBV) theory can provide a better explanation of the interplay of the strategic resources of the organization and the capability to gain a competitive advantage. The indicator system framework we constructed was based on RBV. The paper concludes that only by enhancing a hospital's profitability can the balance of hospital revenue and expenditure be increased, thus reducing financial pressure on hospital operation (7). Simultaneously, the enthusiasm of medical staff can be stimulated with the stabilization of a hospital's operation and development, which in turn will enhance patient satisfaction (19, 20). Moreover, talent recruiting, equipment procurement, performance and salary distribution all benefit from the hospital's own efficient operation and steady income (21). In the end, greater talent will participate in the construction and development of TCM hospitals. The details are shown in Figure 1.

The paper makes three main contributions to the literature. First, to our knowledge, research on the TCM medical service system is still in the exploratory stage. The importance and originality of this study are that it explores the logical relationship between the indicators and the weight in the TCM medical service system. And, this is also the first study to undertake a comprehensive evaluation of the performances of the TCM medical service system in 31 provinces of China. Second, this paper focuses on TCM hospitals, so it expands the research scope of health economics and enriches its research contents. Specifically, our research can produce a batch of research results with strong instructive significance and application value, providing a basis for scientific decision-making. Third, existing evaluation methods tend to be based mainly on the perspective of the total medical service, ignoring medical service per Capita by individuals. And, some new evaluation indicators are added to make the evaluation results more practical and reliable. This article continues with section Introduction which briefly reviews the relevant literature on the TCM medical service system. Section Data and Methods discusses Materials and Methods, while section Empirical Analysis exhibits the empirical analysis of the TCM medical service system. We also illustrate the variables used in medical service system studies. Conclusions and suggestions for future research are presented in section Results.

## Data and Methods

### Data Sources

Data are obtained from the Health Statistics Yearbook from 2013 to 2018 and from the National Statistics of Chinese Medicine from 2012 to 2017. In this paper, TCM hospitals in 31 provinces are selected as the research objects. TCM hospitals are medical institutions that treat patients with TCM service and products to maintain public health (9). Using the Delphi consensus methodology, 12 experts from the field of health-care and reviewing research on hospital performance evaluation from China were asked during three rounds of questioning to score the feasibility and importance of indicators that could be used to determine the framework of the TCM medical service system. After multiple pre-evaluation and expert discussions, 14 indicators have been selected for empirical analysis. Descriptive statistics are shown in Table 1.

**Table 1:** Factor analysis index system.

**Variable**	**Indicator**	**Definitions**	**Observations**	**Mean**	**Std. Dev**.	**Probability**
X1	Number of TCM hospitals per 10,000 people	The number of TCM hospitals divided by the total population.	186	12.60	16.60	0.00
X2	Actual number of open beds per 1,000 people	The number of beds that can be used for every thousand people. Number of beds currently owned by Chinese medicine hospitals at the end of the year.	186	22.68	26.46	0.00
X3	Number of TCM doctors per 1,000 people	Number of TCM doctors per 1,000 people. Those who have obtained the certificate of physician practitioner (assistant) and are currently engaged in medical treatment and preventive health care.	186	13.59	16.24	0.00
X4	Number of practicing TCM doctors per 1,000 people	Number of trainee Chinese medical doctors per thousand people. An intern who has received a medical diploma but has not yet received a medical license.	186	0.45	0.62	0.00
X5	Number of herbalists per 1,000 people	The number of TCM pharmacists per thousand people, who have obtained the certificate of licensed TCM pharmacist and are engaged in the dispensing, preparation, verification and production of medicines in medical institutions.	186	4.07	6.03	0.00
X6	Medical income (mil CNY)	Medical income refers to the income obtained by medical institutions in carrying out medical service activities.	186	24561.08	75811.35	0.00
X7	Medical operational expenditure (mil CNY)	Medical expenses refer to the expenses incurred by medical and health institutions in providing medical services and supporting activities.	186	23426.36	74211.74	0.00
X8	Outpatient treatment costs per patient (CNY)	Total outpatient expenditure divided by the number of outpatients.	186	155.34	84.54	0.07
X9	Hospitalization costs per inpatient (CNY)	The average annual cost incurred by all inpatients.	186	5556.13	3468.07	0.01
X10	Number of outpatient and emergency visits	The total number of people treated in TCM medical and health institutions.	186	1196.30	1395.94	0.00
X11	Number of discharged patients	The number of people discharged from a hospital within 1 year.	186	506522.40	535931.70	0.00
X12	Bed occupancy rate (%)	Actual total occupied bed days divided by actual open from bed days.	186	84.62	8.29	0.02
X13	Average length of stay in hospital (day)	Total number of days in bed occupied by discharged patients divided by number of discharged patients.	186	10.12	1.00	0.00
X14	Daily inpatients per doctor	Total number of beds actually occupied per day divided by the average number of physicians.	186	3.30	2.59	0.00

### Research Methods

Exploratory Factor Analysis (EFA) is a statistical approach for determining the correlation among the variables in a data set (22). This type of analysis provides a factor structure (a grouping of variables based on strong correlations). A critical assumption of the EFA is that it is only appropriate for sets of non-nominal items that theoretically belong to reflective latent factors. Based on the analysis of the correlation coefficient matrix of variables, data reduction factor analysis conducts dimensionality reduction processing on existing data in order to reflect the most information from the original variables with fewer indicators (23). Therefore, the problem studied can describe each component of the original observation through the sum of the linear function of the least number of common factors and special factors.

**Figure 2:**
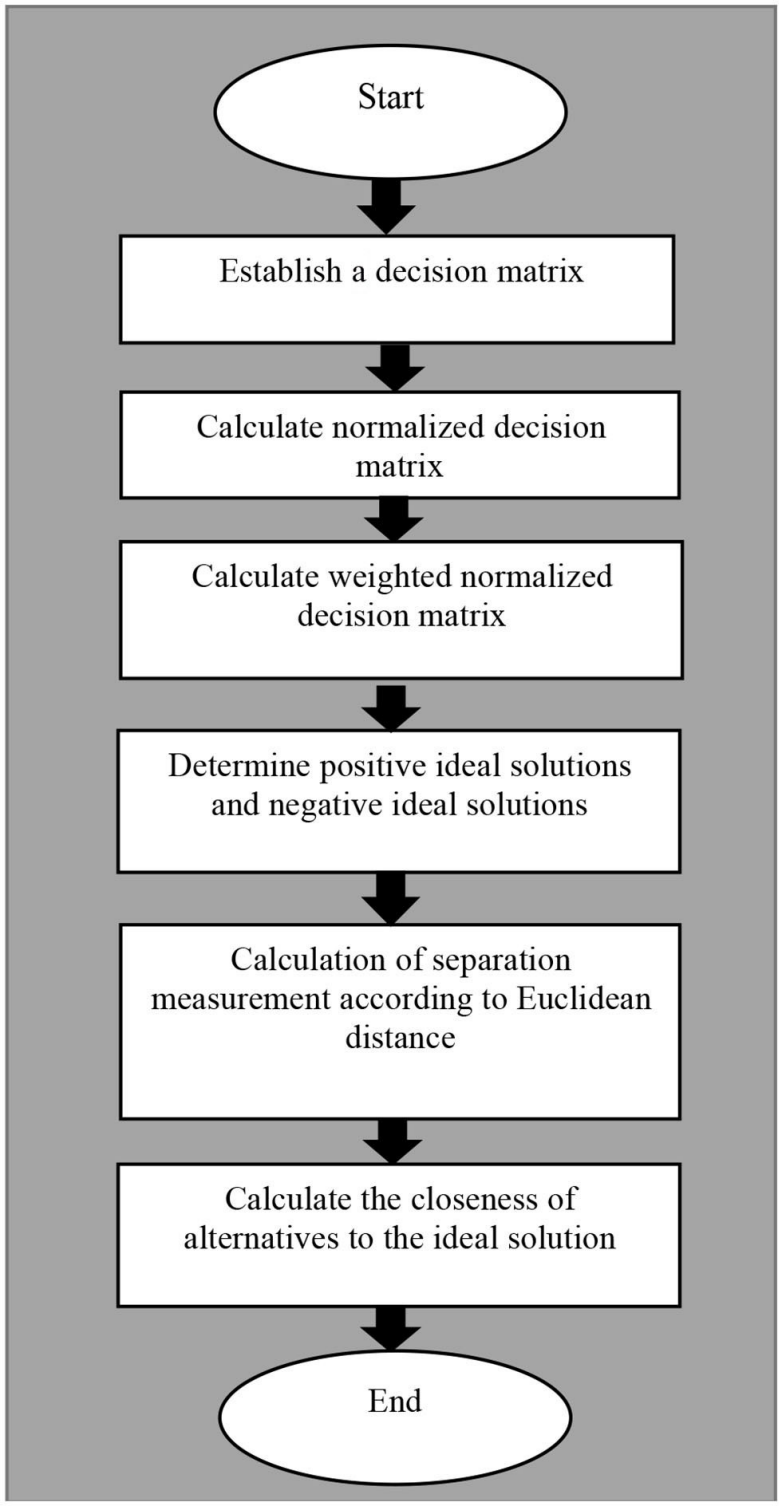
The ranking scheme for integrated TOPSIS.

The Technique for Order of Preference by Similarity to Ideal Solution (TOPSIS) is a multi-criteria decision analysis method to identify solutions from a finite set of alternatives based upon simultaneous minimization of distance from an ideal point and maximization of distance from a nadir point (24). According to the degree to which the evaluation object and the idealized targets are close to each other, if a feasible solution is the closest to the ideal solution and the furthest away from the negative ideal solution, it means that the evaluation object is closer to the optimal level. The ranking scheme for integrated TOPSIS is shown in Figure 2 and described as follows.

### Theory Model

#### Factor Analysis

**Step 1:** Construction of simultaneous equations

Suppose there are *m* test variables *X*_1_*, X*_2_*, …, X*_*m*_ that contain p independent common factors *F*_1_*, F*_2_*, …, F*_*p*_ (*m* ≥ *p*), test variable *X*_*i*_ contains unique factor *U*_*i*_ *(i* = *1,2, …,m)*, every *U*_*i*_ is not related to each other, and they are uncorrelated to *F*_*i*_ *(j* = *1,2, …,p)*. Each *X*_*i*_ can be linearly expressed by *p* common factors and its corresponding unique factor *U*_*i*_, which is provided by the following:

(1) $$\begin{array}{l} \{\begin{array}{c} X_{1}=a_{11}F_{1}+a_{12}F_{2}+\cdot \cdot \cdot +a_{1p}F_{p}+c_{1}U_{1}\\ X_{2}=a_{21}F_{1}+a_{22}F_{2}+\cdot \cdot \cdot +a_{2}F_{p}+c_{2}U_{2}\\ \cdot \cdot \cdot \cdot \cdot \cdot \cdot \cdot \cdot \cdot \cdot \cdot \cdot \cdot \cdot \cdot \cdot \cdot \cdot \cdot \cdot \cdot \cdot \cdot \cdot \cdot \cdot \cdot \cdot \cdot \cdot \cdot \cdot \cdot \cdot \\ X_{m}=a_{m1}F_{1}+a_{m2}F_{2}+\cdot \cdot \cdot +a_{mp}F_{p}+c_{m}U_{m} \end{array} \end{array}$$

**Step 2:** Construct the normalized decision matrix

In this process, the decision matrix is provided by the following:

(2) $$\begin{array}{l} \left(\begin{array}{c} X_{1}\\ X_{2}\\ \cdot \cdot \cdot \\ X_{m} \end{array}\right)={\left(a_{ij}\right)}_{m\times p}\cdot \left(\begin{array}{c} F_{1}\\ F_{2}\\ \cdot \cdot \cdot \\ F_{p} \end{array}\right)+\left(\begin{array}{c} c_{1}U_{1}\\ c_{2}U_{2}\\ \cdot \cdot \cdot \\ c_{m}U_{m} \end{array}\right) \end{array}$$

$$\begin{array}{l} \underset{m\times 1}{X}=\underset{m\times p}{A}*\underset{p\times 1}{F}+\underset{m\times 1}{C}\underset{m\times 1}{U} \end{array}$$

subject to:

(I) p ≤ m;

(II) COV(F· U) = 0 (*F* is irrelevant to *U*);

(III) E(F) = 0 COV(F) = ${\left(\begin{array}{lll} 1 & \ddots & 1 \end{array}\right)}_{p\times p}=I_{p}$= *I*_*p*_.

Common factors *F*_1_*, F*_2_*, …, F*_*P*_ are not correlated, and the variances and the means are both 1 and 0.

(IV) E(U) = 0 COV(U) = I_m_

*U*_1_*, U*_2_*,…,U*_*m*_ are irrelevant and they are both normalized variables. Assume that *X*_1_*, X*_2_*,…, X*_*m*_ are standardized, but not independent of each other. *A* is called factor loading matrix, and its element *a*_*ij*_ represents the load of the *i-th* variable *X*_*i*_ on the *j-th* common factor *F*_*j*_, which is referred to as factor load. If *X*_*i*_ is regarded as a vector in the factor space of *P* dimension, *a*_*ij*_ represents the projection of *X*_*i*_ on the coordinate axis *F*_*j*_. The purpose of factor analysis is to replace *X* with *F* through the model (1) or (2). Generally, *p* < *m* is used to simplify the dimension of variables.

#### TOPSIS

**Step 1:** Establish a decision matrix.

Suppose the decision matrix is *A*.

(3) $$\begin{array}{l} A=\left[\begin{array}{cccc} f_{11} & f_{12} & \cdots & f_{1m}\\ f_{21} & f_{22} & \cdots & f_{2m}\\ \vdots & \vdots & \cdots & \vdots \\ f_{n1} & f_{n2} & \cdots & f_{nm} \end{array}\right] \end{array}$$

**Step 2**: Calculate normalized decision matrix.

Normalized decision matrix *Z*′ is formed by decision matrix *A* and its elements are $Z_{\;ij}^{\prime }$, which is shown below.

(4) $$Z_{ij}^{\;\prime }=\frac{f_{ij}}{\sqrt{\sum_{i=1}^{n}f_{ij}^{2}}}$$

**Step 3**: Calculate weighted normalized decision matrix.

Construct a normalized weighted decision matrix *Z*, and its elements are *Z*_*ij*_.

(5) $$Z_{ij}=W_{j}Z_{ij}^{\;\prime },i=1,\cdots ,n;\;j=1,\cdots ,m$$

**Step 4**: Determine positive ideal solutions and negative ideal solutions.

(6) $$\begin{array}{l} Z^{+}=\left(Z_{1}^{+},Z_{2}^{+},\cdots ,Z_{m}^{+}\right)=\left\{\underset{i}{\max}Z_{ij}|j=1,2,3\cdots ,m\right\} \end{array}$$

(7) $$\begin{array}{l} Z^{-}=\left(Z_{1}^{-},Z_{2}^{-},\cdots ,Z_{m}^{-}\right)=\left\{\underset{i}{\min}Z_{ij}|j=1,2,3\cdots ,m\right\} \end{array}$$

**Step 5**: Calculation of separation measurement according to Euclidean distance.

The Euclidean norm is used as the measure of distance, and the distance from any feasible solution *Z*_*i*_ to *Z*^+^ is:

(8) $$\begin{array}{l} D_{i}^{+}=\sqrt{\overset{m}{\underset{j=1}{\sum }}{\left(Z_{ij}-Z_{j}^{+}\right)}^{2}}\;i=\left(1,2,\cdot \cdot \cdot ,n\right) \end{array}$$

where, *Z*_*ij*_ is the normalized weighted value of the *j-th* target to the *i-th* scheme (solution).

Similarly, if $Z^{-}={\left(Z_{1}^{-},Z_{2}^{-},\cdot \cdot \cdot Z_{m}^{-}\right)}^{T}$is the negative ideal solution of the normalized weighted target, the distance between any feasible solutions and the negative ideal solutions is:

(9) $$\begin{array}{l} D_{i}^{-}=\sqrt{\overset{m}{\underset{j=1}{\sum }}{\left(Z_{ij}-Z_{j}^{-}\right)}^{2}}\;i=\left(1,2,\cdot \cdot \cdot ,n\right) \end{array}$$

**Step 6**: Calculate the closeness of alternatives to the ideal solution.

Then, the relative proximity of a feasible solution to an ideal solution is defined as:

(10) $$\begin{array}{ll} C_{i}^{}=\frac{D_{i}^{-}}{D_{i}^{-}+D_{i}^{+}}\; & 0\leq C_{i}\leq 1,i=\left(1,2,\cdots ,n\right) \end{array}$$

Therefore, *Z*_*i*_ is closer to the ideal solution, *C*_*i*_ is closer to 1 accordingly. On the contrary, *Z*_*i*_ is closer to the negative ideal solution, *C*_*i*_ is closer to 0 accordingly. Then, *C*_*i*_ values of each evaluation object can be compared to obtain a satisfactory solution (25–29).

## Empirical Analysis

### Factor Analysis

**Figure 3:**
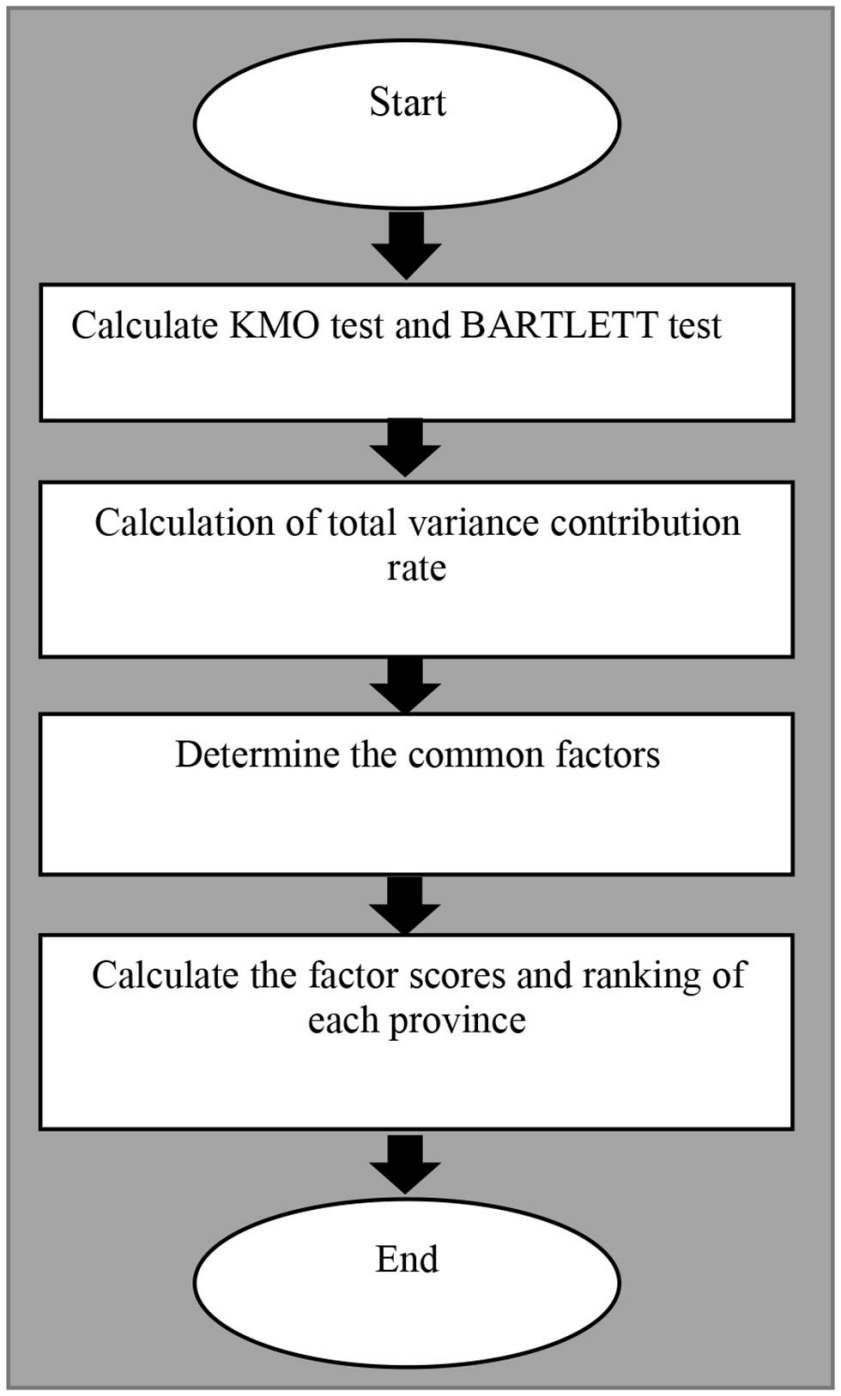
The measurement steps of factor analysis.

The measurement steps of factor analysis are shown in Figure 3 and described as following.

#### KMO Test and Bartlett Test

The Kaiser-Meyer-Olkin (KMO) Test is a measure of how suited data is for Factor Analysis. Bartlett's test for homogeneity of variances is used to test that variances are equal for all samples (30). The selected indicators were analyzed by principal component analysis, as shown in Table 2. The results showed that the KMO were all greater than the minimum standard of 0.5, and most of them were more than 0.6, which was suitable for factor analysis. The Bartlett Test of Sphericity rejected the null hypothesis of the unit correlation matrix, *p* < 0.001.

**Table 2:** KMO test and Bartlett test of cross-section data from 2012 to 2017.

**Year**	**Kaiser-Meyer-Olkin measure of sampling adequacy**	**Bartlett's test of sphericity**		
		**Approx. Chi-Square**	**Df**	**sig**.
2012	0.663	462.103	91	0.000
2013	0.570	479.496	91	0.000
2014	0.691	498.978	91	0.000
2015	0.637	494.996	91	0.000
2016	0.663	524.326	91	0.000
2017	0.692	514.104	91	0.000

#### Total Variance Contribution Rate

The total variance contribution rate of the cross-sectional data factor analysis method from 2012 to 2017 is shown in Table 3. The results show that the characteristic value of the first four principal components is >1, and their cumulative contribution rate is above 80%, indicating that the four common factors cover more than 80% of the secondary index information, with a high degree of interpretation. Therefore, the first four common factors are selected.

**Table 3:** Total variance contribution rate of cross- section data factor analysis method from 2012 to 2017.

**Year**	**Component**	**Initial eigenvalues**			**Extraction sums of squared loadings**			**Rotation sums of squared loadings**		
		**Total**	**% of variance**	**Cumulative %**	**Total**	**% of variance**	**Cumulative %**	**Total**	**% of variance**	**Cumulative %**
2012	1	5.549	39.635	39.635	5.549	39.635	39.635	4.824	34.455	34.455
	2	2.902	20.729	60.365	2.902	20.729	60.365	3.000	21.431	55.886
	3	1.976	14.114	74.479	1.976	14.114	74.479	2.081	14.863	70.749
	4	1.356	9.683	84.162	1.356	9.683	84.162	1.878	13.413	84.162
2013	1	5.435	38.821	38.821	5.435	38.821	38.821	5.083	36.309	36.309
	2	3.123	22.308	61.128	3.123	22.308	61.128	2.872	20.518	56.827
	3	1.947	13.904	75.032	1.947	13.904	75.032	2.373	16.947	73.774
	4	1.037	7.408	82.44	1.037	7.408	82.44	1.213	8.666	82.44
2014	1	5.775	41.25	41.25	5.775	41.25	41.25	5.069	36.207	36.207
	2	3.198	22.845	64.094	3.198	22.845	64.094	3.166	22.611	58.818
	3	1.883	13.448	77.543	1.883	13.448	77.543	2.021	14.433	73.252
	4	1.048	7.487	85.03	1.048	7.487	85.03	1.649	11.778	85.03
2015	1	5.578	39.841	39.841	5.578	39.841	39.841	4.877	34.835	34.835
	2	3.381	24.149	63.99	3.381	24.149	63.99	3.323	23.737	58.572
	3	1.99	14.213	78.203	1.99	14.213	78.203	2.043	14.593	73.165
	4	1.076	7.685	85.888	1.076	7.685	85.888	1.781	12.723	85.888
2016	1	5.611	40.081	40.081	5.611	40.081	40.081	5.118	36.559	36.559
	2	3.431	24.509	64.59	3.431	24.509	64.59	3.239	23.137	59.696
	3	1.67	11.925	76.516	1.67	11.925	76.516	1.957	13.982	73.678
	4	1.265	9.035	85.551	1.265	9.035	85.551	1.662	11.873	85.551
2017	1	5.638	40.274	40.274	5.638	40.274	40.274	4.748	33.913	33.913
	2	3.328	23.769	64.044	3.328	23.769	64.044	3.494	24.959	58.872
	3	1.992	14.23	78.274	1.992	14.23	78.274	2.023	14.448	73.321
	4	1.275	9.11	87.384	1.275	9.11	87.384	1.969	14.064	87.384

#### Common Factors and Explanations

Principal component rotation is a series of mathematical methods to make the component load array easier to explain. We kept the selected components unrelated in order to make the common factor variables reflect the index information clearly. The initial factor loading matrix was rotated by the quartimax method, and the rotated factor loading matrix was obtained. Then, the common factors were named according to the indexes with high load under the common factors after rotation (31). This paper only takes the data of 2017 as an example to illustrate. Table 4 shows that the first common variable F1 has a large load on X6, X7, X8, X9, which can be named as medical income and expenditure. The second common variable F2 has a large load on X1, X2, X3, X4, and X5, which can be named as TCM medical resources. The third common variable F3 has a large load on X10, X11, which can be named as synthetic service capability in hospital. The fourth common variable F4 can be named as service efficiency, because it has a large load on X12, X13, and X14.

**Table 4:** Rotated component matrix in 2017.

**Secondary indicators**	**Component**			
	**1**	**2**	**3**	**4**
Number of TCM hospitals per 10,000 people	(0.041)	0.861	(0.338)	(0.149)
Actual number of open beds per 1,000 people	(0.102)	0.929	0.035	0.197
Number of TCM doctors per 1,000 people	0.394	0.828	0.090	(0.139)
Number of practicing TCM doctors per 1,000 people	0.040	0.519	(0.314)	0.575
Number of Herbalists per 1,000 people	0.489	0.722	0.026	(0.299)
Medical income	0.973	(0.027)	0.124	0.102
Medical operational expenditure	0.972	(0.036)	0.095	0.099
Outpatient treatment costs per patient	0.867	0.260	(0.115)	(0.109)
Hospitalization costs per inpatient	0.959	0.128	0.043	(0.148)
Number of outpatient and emergency visits	0.484	(0.023)	0.789	(0.008)
Number of discharged patients	(0.103)	(0.129)	0.930	0.133
Bed occupancy rate	0.048	(0.246)	0.466	0.752
Average length of stay in hospital	0.382	0.500	0.089	(0.618)
Daily inpatients per doctor	(0.619)	(0.030)	0.222	0.670

#### Factor Scores

The scores of common variables were obtained by using the weighted least squares regression method. In this paper, the 2017 factor score coefficient matrix is obtained by taking the 2017 data as an example (see Table 5). Formula F1, F2, F3, and F4 are the scores of latent variables, which are shown below.

**F1 = −0.059X1−0.077X2+…−0.102X14**

**F2 = 0.249X1+0.327X2+…+0.085X14**

**F3 = −0.082X1+0.095X2+…+0.088X14**

**F4 = −0.012X1+0.138X2+…+0.300X14**

**Table 5:** 2017 component score coefficient matrix.

**Secondary indicators**	**Component**			
	**1**	**2**	**3**	**4**
Number of TCM hospitals per 10,000 people	(0.059)	0.249	(0.082)	(0.012)
Actual number of open beds per 1,000 people	(0.077)	0.327	0.095	0.138
Number of TCM doctors per 1,000 people	0.014	0.249	0.117	(0.020)
Number of practicing TCM doctors per 1,000 people	0.067	0.160	(0.215)	0.416
Number of Herbalists per 1,000 people	0.035	0.194	0.083	(0.100)
Medical income	0.251	(0.066)	(0.042)	0.153
Medical operational expenditure	0.254	(0.072)	(0.058)	0.155
Outpatient treatment costs per patient	0.202	0.005	(0.109)	0.059
Hospitalization costs per inpatient	0.215	(0.031)	(0.035)	0.018
Number of outpatient and emergency visits	0.048	0.035	0.405	(0.065)
Number of discharged patients	(0.093)	0.064	0.512	(0.071)
Bed occupancy rate	0.070	(0.009)	0.130	0.381
Average length of stay in hospital	(0.018)	0.122	0.153	(0.322)
Daily inpatients per doctor	(0.102)	0.085	0.088	0.300

“*()” denotes that the indicator is a negative value*.

The scores of common factors are obtained by using the weighted least squares regression method (32). According to the results of the component score coefficient matrix, it is easy to obtain the common variables' scores and the ranking of TCM medical service system in 31 provinces from 2012 to 2017, as shown in Table 6. According to the scores of each common variable, the variance contribution rate of each latent variable is used as the weight. The ratio between the variance contribution rate of each common variable and the cumulative contribution rate is used as the latent variable value coefficient (33, 34). The comprehensive factor scores F of 31 provinces are calculated, and F reflects the development of TCM medical services in 31 provinces.

**F = 0.388F1+0.286F2+0.165F3+0.161F4**

**Table 6:** China's comprehensive factor score and ranking of 31 provinces from 2012 to 2017.

**Province**	**2012**		**2013**		**2014**		**2015**		**2016**		**2017**	
	**Score**	**Ranking**	**Score**	**Ranking**	**Score**	**Ranking**	**Score**	**Ranking**	**Score**	**Ranking**	**Score**	**Ranking**
Beijing	1.80	1	2.31	1	2.08	1	2.02	1	2.21	1	2.07	1
Tianjin	0.59	4	0.64	3	0.54	5	0.43	6	0.62	3	0.26	7
Hebei	(0.56)	27	(0.38)	26	(0.47)	27	(0.52)	27	(0.31)	23	(0.37)	26
Shanxi	(0.72)	30	(0.24)	20	(0.56)	28	(0.72)	30	(0.22)	20	(0.69)	30
Inner Mongolia	(0.31)	23	(0.33)	22	(0.07)	17	(0.12)	19	0.21	8	0.02	15
Liaoning	(0.15)	18	(0.39)	27	(0.19)	20	(0.25)	22	(0.00)	13	(0.31)	22
Jilin	(0.62)	28	(0.57)	31	(0.58)	29	(0.59)	29	(0.22)	21	(0.57)	29
Heilongjiang	(0.36)	24	(0.54)	30	(0.34)	23	(0.37)	25	(0.15)	16	(0.49)	28
Shanghai	0.68	3	0.31	7	0.46	6	0.55	4	0.44	4	0.57	3
Jiangsu	0.56	6	0.52	5	0.55	4	0.49	5	0.42	5	0.48	5
Zhejiang	0.81	2	0.68	2	0.82	2	0.70	2	0.75	2	0.67	2
Anhui	(0.41)	25	(0.42)	28	(0.40)	24	(0.35)	23	(0.46)	27	(0.29)	20
Fujian	(0.14)	17	(0.08)	14	(0.16)	19	(0.25)	21	(0.18)	19	(0.31)	21
Jiangxi	(0.22)	20	(0.36)	24	(0.26)	21	(0.25)	20	(0.35)	24	(0.36)	24
Shandong	0.03	12	0.17	8	0.15	10	0.09	12	0.14	9	0.11	10
Henan	(0.01)	13	(0.05)	12	0.02	12	(0.09)	17	0.01	12	(0.07)	17
Hubei	0.09	11	(0.14)	17	0.09	11	0.10	11	(0.04)	14	0.04	14
Hunan	0.24	9	0.10	9	0.19	9	0.12	10	0.09	11	0.08	12
Guangdong	0.43	7	0.45	6	0.29	7	0.17	9	0.38	7	0.23	8
Guangxi	(0.01)	14	(0.00)	11	(0.14)	18	(0.10)	18	(0.39)	25	(0.11)	19
Hainan	(0.63)	29	(0.28)	21	(0.82)	31	(0.82)	31	(0.72)	31	(0.80)	31
Chongqing	0.29	8	(0.06)	13	0.19	8	0.35	7	0.10	10	0.29	6
Sichuan	0.58	5	0.52	4	0.58	3	0.55	3	0.39	6	0.53	4
Guizhou	(0.28)	21	(0.14)	16	(0.27)	22	(0.01)	15	(0.51)	28	0.08	11
Yunnan	(0.30)	22	(0.38)	25	(0.46)	26	(0.36)	24	(0.56)	30	(0.35)	23
Tibet	(0.76)	31	(0.54)	29	(0.70)	30	(0.52)	28	(0.51)	29	(0.45)	27
Shaanxi	(0.04)	15	(0.19)	19	(0.04)	15	(0.07)	16	(0.07)	15	(0.07)	18
Gansu	0.13	10	0.00	10	(0.05)	16	0.08	13	(0.18)	18	0.07	13
Qinghai	(0.43)	26	(0.10)	15	(0.03)	14	0.04	14	(0.30)	22	(0.04)	16
Ningxia	(0.21)	19	(0.36)	23	(0.40)	25	(0.50)	26	(0.43)	26	(0.37)	25
Xinjiang	(0.07)	16	(0.15)	18	(0.00)	13	0.22	8	(0.16)	17	0.14	9

“*()” denotes that the indicator is a negative value*.

By comparing F1 medical income and expenditure, Beijing, Shanghai, Tianjin, Zhejiang, Jiangsu, Guangdong, Shandong, Liaoning, Chongqing, and Fujian rank in the top 10. That means the revenue and expenditure capacity of TCM medical services in North and East China is generally higher than that in Northwest and Southwest China, and the average diagnosis and treatment cost per out-patient and average hospitalization cost per inpatient are also higher than that of Northwest and Southwest China. This is because East China and North China have witnessed rapid economic development compared with Northwest and Southwest China, with increasing disposable income and stronger consumption capacity of residents, which objectively provides an economic foundation for the development of public health services. Meanwhile, East China and North China are also stronger than the Northwest and Southwest in terms of education and international exchange and cooperation, which leads to talent gathering and medical technology upgrading. Eventually, some patients with severe diseases are referred to East China and North China for treatment. In addition, the scale and proportion of medical financial allocation in North China and East China are mostly higher than those in Northwest and Southwest China, which further widens the gap between East and North China and Northwest and Southwest China.

Comparing the F2 TCM medical resources, Beijing, the Inner Mongolia Autonomous Region, Qinghai, Tianjin, Gansu, Zhejiang, the Tibet Autonomous Region, Chongqing, Shanxi, and Sichuan are rich in medical resources per capita, ranking in the top 10. Currently, medical resources can be generally divided into the number of TCM medical service institutions, TCM medical technicians and instruments. Beijing, Tianjin and Zhejiang have gained obvious advantages in medical resources. Because Beijing is the political and economic center of China, its innovation, human capital, and ecology all provide fertile ground for the development of traditional Chinese medicine. Inner Mongolia, Qinghai, Gansu, and Tibet are sparsely populated but ethnic medicine is very advanced. Meanwhile, these provinces also attach great importance to the cultivation of TCM talent. Therefore, per capita allocation of medical resources in Southwest and Northwest regions are stronger than those in Central and South China, but the overall medical resources are weaker than those in East and North China.

Comparing F3 service capability, Sichuan, Guangdong, Jiangsu, Zhejiang, Henan, Shandong, Hubei, Hunan, Hubei, Shanghai, and Anhui rank in the top 10. This indicates that these regions have a prominent performance in the number of visits made by TCM medical institutions and the number of discharges from the hospital of TCM medical institutions. That is to say, they are more capable of providing TCM services than those provinces in Northwest and Southwest China.

Comparing F4 service efficiency, Shanghai, Beijing, Jiangsu, Tianjin, Zhejiang, Guangdong, Shandong, Chongqing, Hubei, and Hunan are at the forefront. This demonstrates that TCM medical service organizations in East China, South China, and Central China have achieved better service results and higher economic benefits with fewer service resources, which is one of the reasons for their relatively strong revenue and expenditure. At the same time, this further explains the efficient allocation of its resources and the balanced operation of the organization.

#### The Changing Trend of China's TCM Medical Service System

**Figure 4:**
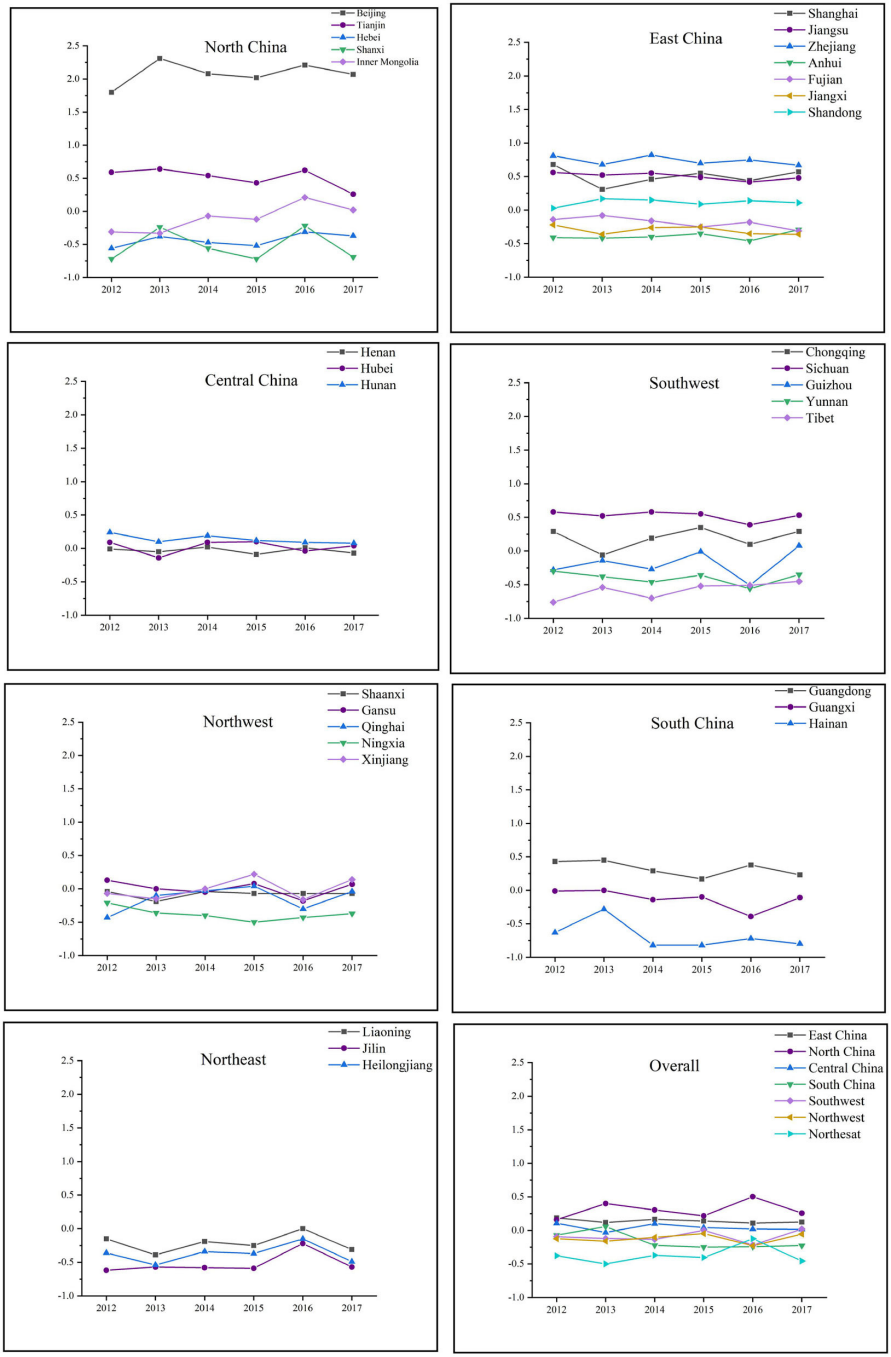
The changing trend of China's TCM medical service system.

Next, from the perspective of regional analysis, the changing trend of China's TCM medical service system from 2012 to 2017 will be analyzed in detail. Figure 4 shows the dynamic fluctuations of TCM medical service systems in seven geographical regions of China. The ranking of the TCM medical service system in each region is North China > East China > Central China > Southwest China > Northwest China > South China > Northeast China. Specifically, Beijing had been far ahead in North China, with Tianjin showing a downward trend, Inner Mongolia and Hebei rising steadily, and Shanxi fluctuating greatly. East China, Central China, and South China were relatively stable, while Southwest China and Northwest China fluctuated greatly on the whole, especially Gansu, Xinjiang, and Qinghai showed an alternating leading. The performance of the TCM medical service system in Northeast China was first increased and then decreased, with Liaoning, Jilin, and Heilongjiang rising consistently since 2013 and reaching a peak in 2016.

The comprehensive factor scores and rankings of China's provinces from 2012 to 2017 are found, but the comprehensive scores of TCM medical services in 31 provinces are different in every year, and the order of comprehensive scores is not the same. This is because the data of each year are independent, so the comprehensive factor scores cannot be simply evaluated by summation. Instead, the results of factor analysis should be comprehensively evaluated by the TOPSIS method. In this way, the issue of factor analysis being unable sum up the panel data to reflect the comprehensive level of a region's TCM medical service system can be solved (35).

### TOPSIS Comprehensive Evaluation

In order to evaluate the development of TCM medical service system of each province from 2012 to 2017 in a more reasonable way, TOPSIS is adopted to process the comprehensive score after factor analysis with MATLAB, so as to obtain the development level and rankings of the TCM medical service system of each province from 2012 to 2017. As shown in Table 7, the comprehensive development level of Beijing, Zhejiang, Sichuan, Tianjin, Shanghai, Jiangsu, Guangdong, and Chongqing is relatively good. The optimal solution proximity in East China and South China is higher than that of Northeast and Southwest China, which is consistent with the above factor analysis results and better reflects the development level of the TCM medical service system in China's 31 provinces.

**Table 7:** Comprehensive evaluation results and rankings of development levels of 2012–2017 in China's 31 provinces.

**Province**	**D+**	**D−**	**Ci**	**Ranking**
Beijing	0.00	2.32	1.00	1
Zhejiang	1.11	1.22	0.52	2
Sichuan	1.28	1.05	0.45	3
Tianjin	1.30	1.04	0.44	4
Shanghai	1.31	1.04	0.44	5
Jiangsu	1.30	1.03	0.44	6
Guangdong	1.45	0.88	0.38	7
Chongqing	1.56	0.80	0.34	8
Hunan	1.60	0.73	0.31	9
Shandong	1.61	0.71	0.31	10
Hubei	1.70	0.65	0.28	11
Gansu	1.71	0.64	0.27	12
Xinjiang	1.72	0.64	0.27	13
Henan	1.74	0.59	0.25	14
Shanxi	1.78	0.56	0.24	15
Inner Mongolia	1.80	0.57	0.24	16
Guangxi	1.82	0.52	0.22	17
Qinghai	1.83	0.53	0.22	18
Guizhou	1.87	0.50	0.21	19
Fujian	1.86	0.46	0.20	20
Liaoning	1.89	0.46	0.20	21
Jiangxi	1.96	0.38	0.16	22
Heilongjiang	2.02	0.34	0.14	23
Ningxia	2.02	0.32	0.14	24
Anhui	2.03	0.31	0.13	25
Yunnan	2.04	0.30	0.13	26
Hebei	2.06	0.27	0.12	27
Shaanxi	2.14	0.22	0.09	28
Jilin	2.14	0.22	0.09	29
Tibet	2.18	0.18	0.08	30
Hainan	2.27	0.10	0.04	31

Therefore, the comprehensive factor score is basically consistent with the result of the optimal solution closeness degree (36). For example, Beijing ranks first in the comprehensive factor scores from 2012 to 2017. Although there is a slight decrease in the comprehensive factor score of Sichuan province from 2012 to 2017, it shows an overall upward trend, and the overall development trend is relatively good, ranking third in the final optimal solution proximity. Therefore, provinces with higher comprehensive factor scores have bigger closeness of alternatives to the ideal solution, and provinces with lower comprehensive factor scores have smaller closeness of alternatives to the ideal solution. For example, the comprehensive factor score of Hainan province from 2012 to 2017 improved in 2013, but was at the bottom of the list for the rest of the year. The score of Hainan province is very low but undergoes great changes, which reflects its slow and unstable development, making the smallest closeness of alternatives to the ideal solution.

Further analysis shows that the closeness of alternatives to the ideal solution in 93.5% of China's provinces is distributed below 0.5, which indicates that the development level of the TCM medical system of provinces in China is still relatively backward, and there is lots of room for development. The proportion where the closeness of alternatives to the ideal solution is bigger than 0.5 only accounts for only 6.5%. The reasons for this may be related to the uneven allocation of resources in the TCM medical service system, a shortage of health technicians and service inefficiency.

## Results

This paper illustrates the evaluation index of China's TCM medical service system and ranks the development of 31 provinces in China.

### TCM Medical Service System

The evaluation index of the TCM medical service system includes TCM medical resources, medical service capability and efficiency, medical income, and expenditure. Doctors and medical equipment are the most important TCM medical resources. They are the technical guarantee for hospitals to provide services. Furthermore, the medical service capacity of TCM hospitals is reflected by the number of patients diagnosed and treated, and the number of patients discharged from hospitals. The service efficiency of TCM hospitals is related to the bed utilization rate, the average hospitalization days and the average hospitalization days for doctors. These factors are the business and economic guarantee of hospital operation. Medical income and expenditure refer to medical service income and medical service cost, which are the foundation of hospital operation and development.

### Ranking of China's TCM Medical Service System

**Figure 5:**
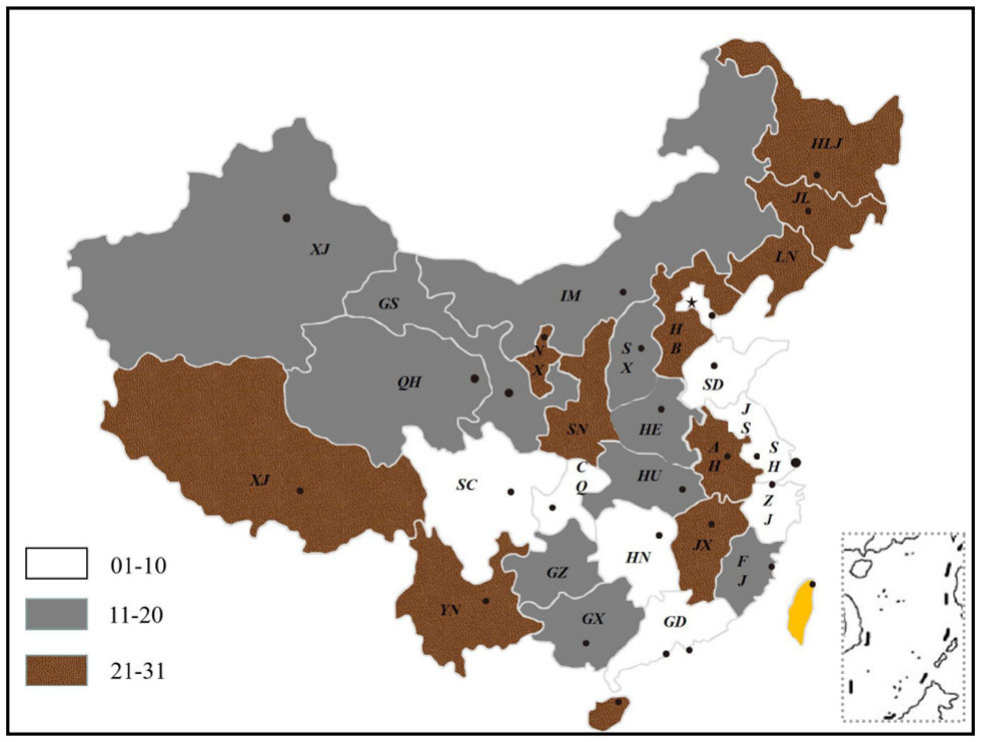
Ranking of China's TCM medical service system.

The paper gives the ranking of 31 provinces in China in terms of the TCM medical service system. It is indicated that Beijing, Zhejiang, Sichuan, Tianjin, Shanghai, Jiangsu, Guangdong, Chongqing, Hunan, and Shandong rank among the top 10 of China. The development of the TCM medical service system in Northwest and Southwest China is weaker than that in East China and Central China. The development of Northeast China tends to be balanced and the strength of each province is close. However, the provinces in North and South China have uneven development and large gaps. This fully reflects the imbalance and inadequacy of China's TCM medical service system in terms of medical resources, service capacity, service efficiency, and medical income and expenditure. To sum up, the development of China's TCM medical service system shows the imbalance and inadequacy of “East is strong, West is weak” and “South is superior, North is inferior.” The details are shown in Figure 5.

## Conclusion and Discussion

This study's results are first aligned with findings from an initial stream of studies that the development of TCM medical service system is unbalanced in China (6). In addition, our study is more comprehensive regarding doctor qualifications, hospital levels and other indicators of TCM service quality. In Lu and Zeng's study, they focused on inequalities in the geographic distribution of hospital beds and doctors in traditional Chinese medicine, while we expand the breadth and depth of the evaluation of China's TCM medical service system. We also enrich the evaluation index of TCM medical resources, for example with the number of Herbalists per 1,000 people. Second, and similar to other prospects (2–4), TCM is a unique health resource in China and one of the main representative traditional medicines globally. Principles for the construction of a comprehensive evaluation of China's TCM medical service system are systematicness, applicability, pertinence, feasibility, and compatibility (7). We have fully considered the systematicness and feasibility of the structure when constructing the TCM medical service system. For example, we explained the internal logic between medical resources, service efficiency, and expenditure-income.

Nonetheless, several limitations of our study should be born in mind. Firstly, our research is based on the perspective of suppliers, and we lack an overview of patient experience of hospital care by a third party. Secondly, although we have made a comprehensive evaluation of the Chinese TCM medical service system, the results fluctuate greatly. We need to explore the essence behind this phenomenon. Therefore, additional research can focus on the performance of hospital services in various provinces or determinants and differences in hospital efficiency.

Based on the above analysis, the paper suggests that the government should increase its support for the TCM medical service system in terms of policies and investment, oversee the training of the next generation of talent in traditional Chinese medicine, improve the teaching quality of colleges and universities of traditional Chinese medicine, and transfer talent from the East to the Central and Western regions so as to enhance the strength of medical resources in these regions (37, 38). Furthermore, the capability of medical services in the Western regions can be improved through counterpart support, medical consortia and telemedicine services. Also, TCM hospitals should strengthen the standardized construction and scientific management of the TCM preventive treatment of diseases section, and improve the service ability of TCM preventive treatment of diseases.

## Data Availability Statement

All datasets generated for this study are included in the article/supplementary material.

## Author Contributions

Z-GL and HW participated in study design, drafted this manuscript, and have all reviewed and approved of the final manuscript. Z-GL conducted data collection and data analysis. All authors contributed to the article and approved the submitted version.

## Conflict of Interest

The authors declare that the research was conducted in the absence of any commercial or financial relationships that could be construed as a potential conflict of interest.

## Abbreviations

TCM: traditional Chinese medicine
KMO: Kaiser-Meyer-Olkin.
